# Collection and Rearing of Container Mosquitoes and a 24-h Addition to the CDC Bottle Bioassay

**DOI:** 10.1093/jisesa/ieaa059

**Published:** 2020-11-02

**Authors:** Casey Parker

**Affiliations:** University of Florida, Institute of Food and Agricultural Sciences, Florida Medical Entomology Laboratory, Vero Beach, FL

**Keywords:** container mosquito, resistance, CDC bottle bioassay, *Aedes*, rearing

## Abstract

Container mosquitoes (Diptera: Culicidae) oviposit their eggs in both natural and artificial containers. Many container mosquito species also serve as important vectors of disease-causing pathogens including *Aedes aegypti*, *Ae. albopictus*, and *Ae. triseriatus*. Control of these species can be done through the use of adulticide sprays. The efficacy of these treatments is highly dependent on the insecticide susceptibility status of the local mosquito populations. This paper provides protocols on collecting and rearing container mosquitoes for use in the Centers for Disease Control and Prevention (CDC) bottle bioassay. A brief description of the CDC bottle bioassay is provided as well as a standardized protocol for the incorporation of a 24-h mortality to the CDC bottle bioassay. Results from this 24-h holding addition to the CDC bottle bioassay reveal that some forms of resistance may be missed without the incorporation of the additional mortality reading. These protocols provide a foundation for new laboratories to establish rearing protocols and begin conducting resistance monitoring.

Container mosquito (Diptera: Culicidae) species oviposit their eggs in natural or artificial containers. In North America, the most common container species includes *Aedes aegypti* (Linnaeus), *Ae. albopictus* (Skuse), *Ae. japonicus* (Theobald), and *Ae. triseriatus* (Say) (Connelly and Rey 2016, Reed et al. 2019). *Aedes aegypti* and *Ae. albopictus* are vectors of dengue (Simmons et al. 2012), chikungunya (Leparc-Goffart et al. 2014), yellow fever (Jentes et al. 2011), and Zika viruses (Likos et al. 2016, McKenzie et al. 2019) and *Ae. triseriatus* is a vector of La Crosse virus (Beaty and Thompson 1975). Little field evidence exists to elucidate *Ae. japonicus*’ role as a major vector, but laboratory studies have shown it to be a competent vector for West Nile, Saint Louis encephalitis, eastern equine encephalitis, and La Crosse viruses (Kaufman and Fonseca 2014). Additionally, it is a highly invasive mosquito that has significantly expanded its range in the United States since its introduction (Andreadis and Wolfe 2010, Schaffner et al. 2013).

Container mosquito species can be difficult to control due to the container larval habitats they occupy. Therefore, control of these invasive and important vectors relies heavily, although not exclusively, on the use of insecticides to control the adult populations. Resistance to insecticides threatens the efficacy of these products and therefore, the ability to control the spread of these mosquitoes and the viruses they may transmit. Insecticide resistance in mosquitoes has been reported globally in 68 countries (World Health Organization 2019) and several studies have documented it in the United States in *Aedes* species specifically (Liu et al. 2004; Marcombe et al. 2014; Cornel et al. 2016; Richards et al. 2017, 2018; Estep et al. 2018). However, many mosquito control programs may not have the ability to monitor for resistance. If they do, the lack of a widely used resistance reporting system means that the information is not widely disseminated to the vector control, public health, and scientific communities. Therefore, increasing the accessibility of resistance monitoring assays to mosquito control programs is critical to monitoring and responding to resistance.

The insecticide susceptibility status of field populations of mosquitoes can be monitored using several methods (Coleman and Hemingway 2007). The Centers for Disease Control and Prevention (CDC) bottle bioassay (Brogdon and McAllister 1998) and the WHO assay (World Health Organization 2018) can detect phenotypic resistance in mosquito populations. Both assays expose the mosquito to an insecticide-treated or -coated surface and monitor mortality over time. While the protocols differ, they tend to agree on the insecticide susceptibility status of populations (Aïzoun et al. 2013). The CDC bottle bioassay can be a cheaper assay to run and offer more flexibility with testing (i.e. obtaining assay materials, addition of a synergist, testing formulated product, etc.). However, only the WHO protocol includes a 24-h mortality reading after the mosquito has been removed from exposure to the insecticide treatment. Richards et al. (2017) noted that while running CDC bottle bioassays, some mosquitoes that were previously categorized as dead would then recover and be counted as ‘alive’, indicating a form of knockdown resistance. Another paper has also pointed out that the lack of a 24-h reading could allow mechanisms, such as metabolic resistance, to be overlooked (Owusu et al. 2015).

Obtaining swift results is considered to be a strength of the CDC bottle bioassay method, but the lack of a 24-h mortality reading can allow the presence of resistant individuals to be missed. Failure to detect recovery in mosquitoes after insecticide exposure means that knockdown resistance in these populations could be overlooked. The optional addition of 24-h reading would address this problem and provide more detailed information on the resistance profile of the mosquito population being tested.

The CDC bottle bioassay can be conducted using field-collected mosquitoes, or by collecting eggs or larvae, and rearing in an insectary. When low numbers of individuals are obtained from the field, populations can be amplified using bloodfeeding. Collection of field populations of container mosquitoes is a necessary first step for phenotypic resistance monitoring, but these methods can also be utilized for other types of assays such as virus infection studies, behavioral assays, and molecular or biochemical assays.

Here, we present standardized methods for collecting container mosquitoes from the field, rearing them in a laboratory, amplifying the populations, and using them for CDC bottle bioassay testing. These methods are optimized for implementation by individual mosquito control programs, but could also be used to investigate resistance over a large geographic area. These methods have been used successfully in a statewide distribution and resistance monitoring program (Parker et al. 2019, 2020) as collected eggs can easily be mailed to a facility where resistance monitoring can take place. Additionally, we present a standardized protocol for the addition of a 24-h mortality reading. The importance and validation of this 24-h addition is also addressed by using field and laboratory populations of mosquitoes. These protocols will be useful to any mosquito control program looking to start a container mosquito surveillance program, resistance monitoring, or any academic or public health institution that wants to launch a statewide/centralized program.

## Experimental Design

For the bottle bioassay, the CDC recommends using populations of mosquitoes collected from the field or collecting eggs or larvae and rearing them in an insectary. *Aedes aegypti* and *Ae. albopictus* exhibit skip oviposition (Colton et al. 2003, Davis et al. 2015) lay their eggs singly, in small containers. Therefore, collecting large numbers of eggs or larvae from the field can be challenging without the use of ovicups. Field-collected adults may also be used directly in the bottle bioassay, but their physiological status must be determined and accounted for (CDC 2020).

Field populations of mosquitoes that are no greater than two generations from the field (F2) as the CDC MosquitoNet system (https://wwwn.cdc.gov/Arbonet/MosquitoNET/) does not accept resistance data collected on populations greater than F2. The successive bloodfeeding of mosquitoes can alter the resistance levels that are detected in the CDC bottle bioassay (CDC 2016). The population of mosquitoes that is collected from the field is the F0 population and the eggs the F0 population produces after bloodfeeding are the F1 population. Subsequent hatching, rearing, and bloodfeeding of the F1 population will result in the F2 population. Collection of container mosquito eggs can be done simply and cheaply as these mosquitoes generally oviposit in natural and artificial containers. Once the mosquito eggs are collected, they can be quantified, hatched, reared, and amplified for use in the CDC bottle bioassay.

The CDC bottle bioassay is a tool that can assess the susceptibility status of mosquito populations to various active ingredients (AIs). AIs are the active/killing agent within insecticides and do not contain other components of a formulated product, such as inert ingredients. The CDC recommends using pure AI and not formulated product for the CDC bottle bioassay because underlying resistance can be masked. The current CDC bottle bioassay lacks a 24-h mortality reading, which can provide additional details on the resistance profile of field populations of mosquitoes. Materials needed for each of these techniques are detailed in Table 1.

**Table 1.** List of materials needed to collect mosquitoes from the field, rear them in a laboratory setting, conduct a CDC bottle bioassay, and incorporate a 24-h mortality reading to the CDC bottle bioassay

Item name	Product number	Link	Use
16-oz black plastic cups	STDMCUP02	https://www.promotionchoice.com/products/16oz-Stadium-Cups.html	Field collection of eggs
Germination paper	400HPT	https://seedburo.com/products/3364	Field collection of eggs
Binder clips	831602	https://www.staples.com/Staples-Medium-Metal-Binder-Clips-Black-1-1-4-Size-with-5-8-Capacity/product_831602?akamai-feo=off	Field collection of eggs
Handheld hole punch	273727	https://www.staples.com/Charles-Leonard-1-Hole-Paper-Punch-With-Metal-Catch/product_273727	Field collection of eggs
Dissecting microscope			Laboratory handling and rearing
Larval rearing trays	1426	https://www.bioquip.com/Search/DispProduct.asp?pid=1426A	Laboratory handling and rearing
Rearing tray lids	1426AC or 1426BC	https://www.bioquip.com/Search/DispProduct.asp?itemnum=1426AC	Laboratory handling and rearing
Adult mosquito cage	DP1000	https://shop.bugdorm.com/bugdorm-1-insect-rearing-cage-p-1.html	Laboratory handling and rearing
Mechanical aspirator	2809B	https://www.bioquip.com/search/DispProduct.asp?pid=2809B	Laboratory handling and rearing
Mouth aspirator with HEPA filter	Model 612	https://www.johnwhock.com/products/aspirators/mouth-aspirators/	Laboratory handling and rearing
Disposable transfer pipettes	1216H30 or 1216H33	https://www.thomassci.com/Laboratory-Supplies/Transfer-Pipets/_/Disposable-Transfer-Pipets?q=Plastic%20Pipettes	Laboratory handling and rearing
Small jar or container (~120 ml)	G850CL	https://www.containerandpackaging.com/products/92/glass-straight-sided-jar/G850CL	Laboratory handling and rearing
Emergence container	1425	https://www.bioquip.com/Search/DispProduct.asp?itemnum=1425	Laboratory handling and rearing
Larval diet—1:1 ratio by weight Brewer’s yeast and lactalbumin			Laboratory handling and rearing
10% sucrose solution			Laboratory handling and rearing
Cotton balls			Laboratory handling and rearing
250-ml Wheaton bottles	219417	https://wheaton.com/250-ml-btl-media-clr-type-i-no-cap.html	CDC bottle bioassay
Screw lids for bottles	240080	https://wheaton.com/33-430-cap-phen-blk-ldpe-lnr.html	CDC bottle bioassay
Micropipettes and disposable tips			CDC bottle bioassay
Amber bottles		https://www.containerandpackaging.com/products/12/glass-boston-round/G149	CDC bottle bioassay
Timer			CDC bottle bioassay
Labeling tape	89098062	https://www.thomassci.com/acs/packaging/packaging-tapes/acs-packaging-tapes-industrial/_/General-Purpose-Laboratory-Labeling-Tape-Rainbow-Pack?q=Laboratory%20Labeling%20Tape	CDC bottle bioassay
Chemical-resistant gloves	19-148	https://www.fishersci.com/shop/products/ansell-touchntuff-disposable-chemical-resistant-nitrile-gloves/p-5349229	CDC bottle bioassay
Insecticide		https://www.chemservice.com/ OR https://www.sigmaaldrich.com/technical-documents/articles/analytical/pesticides.html	CDC bottle bioassay
Formulated product			CDC bottle bioassay
Acetone			CDC bottle bioassay
Lab soaker paper	62050	https://www.thermofisher.com/order/catalog/product/62050-00	CDC bottle bioassay
8-oz paper food cups	760SOUP8WPA	https://www.webstaurantstore.com/choice-8-oz-double-poly-coated-white-paper-soup-hot-food-cup-with-vented-paper-lid-case/760SOUP8WPA.html	24-h CDC bottle bioassay addition
Tulle or mesh	16063042	https://www.joann.com/casa-collection-solid-tulle-fabric-57/zprd_16057598a.html	24-h CDC bottle bioassay addition

Item name, product (or catalog) number, a link, and the items use have been included here.

### Field Collection of Eggs

**Fig. 1.**
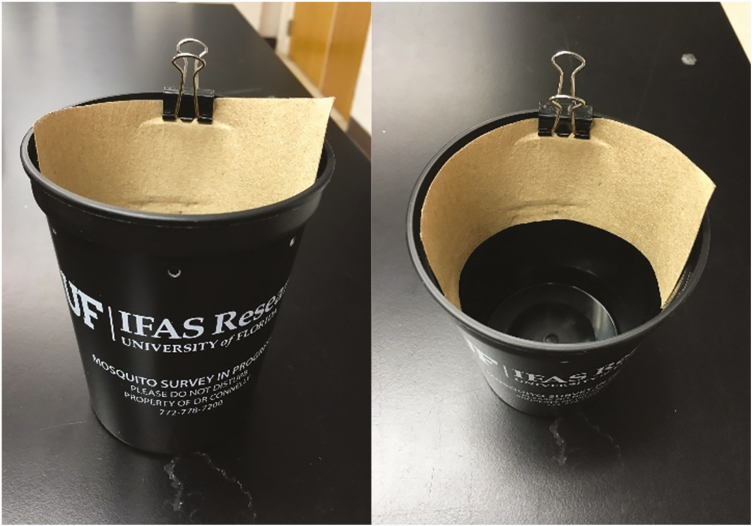
Basic ovicup made from a black plastic cup, binder clip, and germination paper.

A 16-oz black plastic cup, germination paper, binder clips, and a handheld hole punch are all that are needed to create an ovicup (Fig. 1). The hole punch should be used to punch at least one hole approximately 1 inch from the top of the cup. This hole prevents the ovicup from overfilling with water, which would prevent oviposition by container mosquitoes. The germination paper should be cut to fit along the inside rim of the cup. The germination paper can then be secured using a binder clip. The number of ovicups needed to collect a sufficient number of container mosquitoes will be dependent on the area of collection as well as the target species. However, preparing 10–30 ovicups per collection area is an ideal starting point.

The germination paper secured on the inside of the ovicup will act as a substrate for container mosquitoes to oviposit on. Enough water to fill all ovicups should be brought into the field. Using a leaf or hay infusion in the ovicups can be more attractive for ovipositing mosquitoes (Reiter et al. 1991, Ponnusamy et al. 2010), but it is not necessary to collect eggs. Ovicups will accumulate organic debris, like leaves, naturally once they are deployed in the field. In areas where *Ae. aegypti* and *Ae. albopictus* are present, it is likely that these two species will make up a majority of the eggs collected. However, other species may utilize ovicups for oviposition.

When using ovicups, it is beneficial to deploy several within an area. *Aedes aegypti* and *Ae. albopictus* exhibit a behavior known as skip oviposition and will oviposit one clutch of eggs in several containers (Colton et al. 2003, Davis et al. 2015). Additionally, these two species oviposit in a wide variety of containers and the ovicup will likely be one of many locations where the mosquitoes will lay their eggs. Deploying several ovicups in one area will increase changes of collecting enough eggs for the assays.

The number of ovicups used in an area will depend on the area, but some examples are included here. In a neighborhood where you have a cluster of houses on a street or in a block, placing approximately 2–3 ovicups at 5–10 houses is a good starting point. In a cemetery, 10–20 ovicups can be randomly distributed around the premises. It is important to note that many container mosquitoes have a short flight range of usually no more than 800 m (Honorio et al. 2003). Therefore, when placing ovicups, the eggs collected should only be considered as the same ‘population’ if they are within this 800 m range. Ovicups should be placed in a location that is shaded and protected. It is best to pick a site that will minimize exposure to animals and curious passersby. An example would be underneath a bush where it is not readily seen by people or animals or easily run over by a lawnmower.

**Fig. 2.**
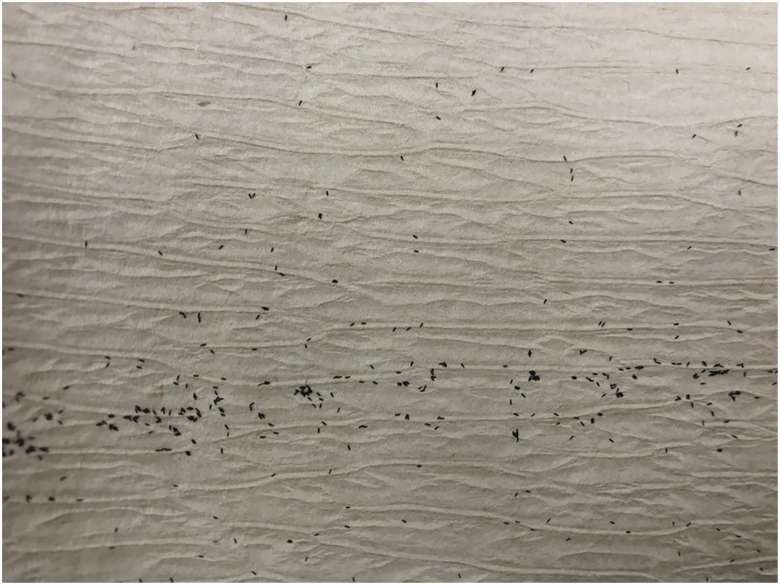
*Aedes* mosquito eggs on germination paper.

After the ovicups are placed and filled with water, they should be monitored every 5 d. This will minimize the number of newly laid eggs that are able to hatch and will ensure that the ovicup does not become a producer of adult mosquitoes in the area. When servicing the ovicup, check the germination paper for eggs (Fig. 2) and inspect the water for larvae. The egg paper should be replaced even if there are no eggs on the paper as the germination paper will deteriorate over time. If there are eggs on the germination paper, remove it from the cup, let the water drain off of the paper, and place it in a labeled plastic bag. Place a new piece of germination paper in the ovicup and secure it with the binder clip. If there are larvae present in the water, empty the ovicup and refill it with fresh water. When setting and collecting ovicups, be sure to record the date the cup was set, collected, the longitude and latitude (or nearest street address) as well as the habitat type.

The egg paper that was collected from the field will likely be wet when it is brought to the laboratory. The papers should be dried before they are stored. To do this, lay the wet egg paper on a dry paper towel and allow it to air-dry until it is slightly damp. Do not allow the egg paper to become too dry or crispy as this will increase the likelihood of the eggs on the paper desiccating.

Once the papers have been dried, they should be transferred to a new container. This can be a plastic bag, a food storage container (plastic or glass), or another container with a lid. Each population should be in a separate container to prevent any cross-contamination between sites. Lay the egg paper in the container and add a moist cotton ball, but do not allow the cotton ball to touch the egg paper. Replace the lid on the container, but do not completely seal it. This setup will create a humid environment for storing the eggs until they are ready to be hatched. Alternatively, if a humidity chamber is available, it can be used instead and should have an approximate RH of 80 ± 5%.

At least 1 wk should elapse between drying the egg paper and attempting to hatch the eggs. Additionally, it may take several weeks of field collections to obtain enough F0 eggs or testing. A minimum of 50, but ideally 250, viable eggs should be obtained for a population. Because *Ae. aegypti* and *Ae. albopictus* skip oviposit, collecting >50 eggs will improve the genetic diversity of your sample as eggs will have been laid by different individuals. All eggs should be collected within as narrow of a time frame as possible. Some resistance mechanisms, such as *kdr*, can be the result of a single nucleotide polymorphism (Saavedra-Rodriguez et al. 2007, Yanola et al. 2011), and can therefore appear and rapidly increase in frequency throughout a population. Therefore, mosquitoes collected within a narrow time frame will be more genetically similar, offering more meaningful results from resistance assays. Once you have collected enough eggs from the site of interest, ovicups should be removed from the field to prevent them from becoming a larval habitat.

### Laboratory Handling and Rearing

Rearing container mosquitoes requires a climate-controlled rearing room where temperature, relative humidity, and the light:dark cycle can be adjusted. Ideal insectary conditions are a temperature of 27 ± 2°C, RH of 75 ± 5%, and a light:dark cycle of 14:10 h for Florida populations of mosquitoes. These conditions allow for the most consistent results but can be modified to fit the needs of your laboratory, which has been demonstrated by several studies (Gaffigan and Pecor 1997, Imam et al. 2014, Bei Resources 2016, Kauffman et al. 2017, Ote and Kanuka 2018). Adjusments may be necessary for mosquitoes colleted in other geographic regions. Additionally, ensuring the rearing room is free of pests (such as ants) or any fungal growth is necessary to maintain mosquito colonies.

Depending on an organization’s policies, Institutional Animal Care and Use Committee (IACUC) approval or similar certification will be needed if live animals will be used for bloodfeeding. Similarly, certain equipment or facilities may be necessary if using an artificial feeding system and harvested blood. A summary of different bloodfeeding techniques can be found in Bei Resources (2014). Research into your organization’s policies regarding this will be necessary before any bloodfeeding of mosquitoes can take place. Alternatively, if your organization is not able to implement bloodfeeding protocols, a larger collection of eggs will be needed to perform the CDC bottle bioassay. To achieve this, additional ovicups can be placed in field site or the collection window can be slightly extended to allow for additional egg collections.

### CDC Bottle Bioassay

The CDC bottle bioassay is conducted with adult mosquitoes to assess their susceptibility status to different AIs. It can be conducted in most laboratories at room temperature. Acetone is used to dilute the insecticides to the appropriate concentration and coat the inside of bottles used in the assay. Therefore, if a fume hood is available, it should be used when preparing chemicals and coating bottles. If no fume hood is available, bottles should be prepared in a nonconfined and well-ventilated space. It is also recommended to pre-label the bottles that will be used during the CDC bottle bioassay using labeling tape.

### 24-Hour Bottle Bioassay Addition

The lack of a 24-h reading for the CDC bottle bioassay can be considered a shortcoming of this assay as it does not consider recovery after insecticide exposure. Others have addressed this issue by creating a 24-h addition to the CDC bottle bioassay using inexpensive and easily obtained materials (Bagi et al. 2015, Denlinger et al. 2016, Mendoza 2016). The only materials needed are 8-oz paper food cups with lids and tulle or other generic mesh (mesh size less than 800 microns to prevent mosquitoes from escaping).

**Fig. 3.**
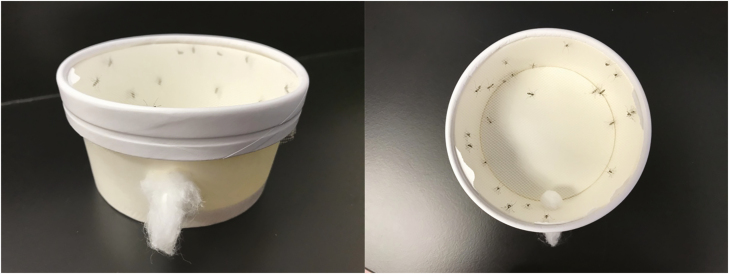
Clean holding cage created by modifying a paper food cup.

The paper food cups (Fig. 3) can be used as clean holding cups for use after the CDC bottle bioassay. To prepare these holding cups, punch a hole in the side. The hole should be large enough for the end of your aspirator to fit into. Either a mechanical or mouth aspirator can be used (Table 1), but the mouth aspirator is preferred because it is less likely to damage the mosquitoes during transfer. The recommended mouth aspirator also incorporates a HEPA filter, so scales from the mosquito are not inhaled. Have a small piece of cotton prepared to plug this hole. Then, take the lid of the cup and remove the center leaving only the outer ring. Cut the mesh or tulle to fit over the holding cup and secure the mesh in place with the modified lid. Prepare as many of these cups as there are bottles being used during the CDC bottle bioassay. The mosquitoes from each bottle will be aspirated into each of these cups. It is best practice to pre-label the holding cups to ensure mosquitoes are released into the correct container.

## Procedure or Protocol

### Laboratory Handling and Rearing

**Fig. 4.**
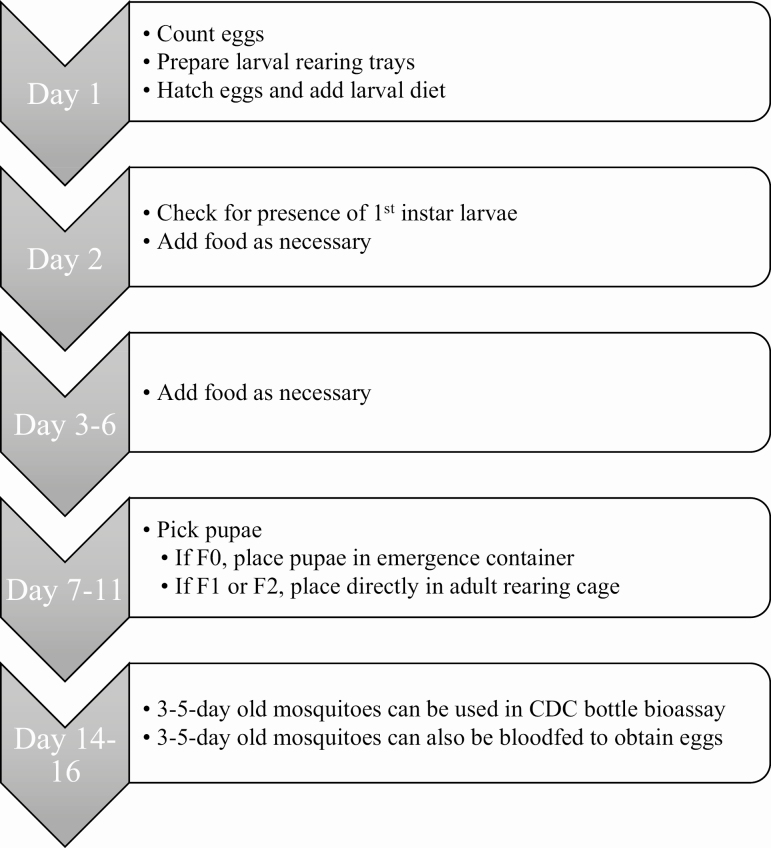
Flow chart of the timing of hatching and rearing container mosquitoes.

**Fig. 5.**
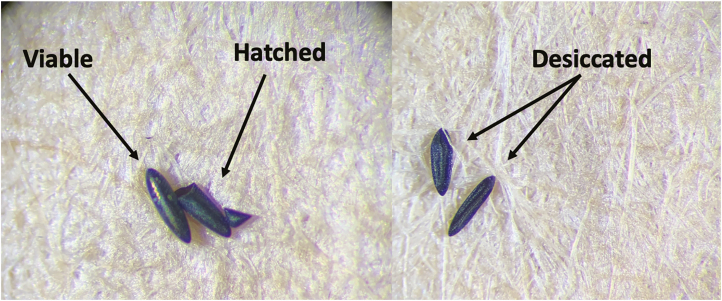
Viable, hatched, and desiccated *Aedes* eggs. Photos provided by Daviela Ramirez.

The length of time from hatching eggs to having 3- to 5-d-old adult mosquitoes (for bloodfeeding or the CDC bottle bioassay) is 14–16 d (Fig. 4). Before eggs are hatched, they should be quantified and classified into categories. This will prevent overcrowding in larval rearing trays and determine if there are enough eggs to begin the rearing and amplification process. Using a dissecting microscope, count all eggs on the paper and classify them as 1) viable, 2) desiccated, or 3) hatched (Fig. 5). Additional examples of these three conditions can be found in Bei Resources (2016), Bibbs et al. (2018), and Mayilsamy (2019).

In preparation for hatching, fill larval trays with water. Two hundred and fifty larvae per liter of water will allow mosquito larvae to complete their development under ideal conditions (Asahina 1964). If working with a smaller rearing tray, the number of larvae per tray can be extrapolated from the number provided.

After water has been added to the rearing trays, egg papers can be placed directly in the water. Hold the egg paper underneath the surface of the water until the paper remains submerged on its own. A variety of larval diets have been explored for use with mosquito rearing that may incorporate carbohydrates, animal proteins, artificial diets, yeast, infusions, or some combination of these components (Asahina 1964, Van Handel 1986, Bond et al. 2017). A brewer’s yeast and liver powder combination have been used successfully at the Florida Medical Entomology Laboratory and provides the protein content necessary for larval maturation (Van Handel 1986). Add larval diet (Table 1) to the water once the eggs are submerged to help induce hatching. The amount of food needed will vary depending on rearing tray size, but a small amount (~0.5–1 g per tray) should be added at the hatching stage. Larval trays can be covered with lids after food has been added and left until the next day.

Rearing trays should be checked the following day for first instar larvae. This can be done by using a flashlight to inspect the rearing tray for movement, which is detectable without magnification. If no larvae are visible, it is possible that another ‘flood’ is necessary to induce hatching. To do this, dry the egg paper as described above, store them for 1 wk, and attempt hatching again. If a vacuum hatch system is available, it can be used to synchronize the hatch, but is not necessary. An example of a vacuum hatch system can be found in Foggie and Achee (2009).

Food will need to be added daily as needed and knowing when to add food and how much is based on the condition of the water. Asahina (1964) details the consequences of providing too little or too much larval diet in rearing trays as well as recommendations for preventing scum formation that may slow larval development. For a density of 250 larvae per liter of water, adding 0.2–0.5 g of larval diet per day will likely be sufficient. When little to no larval diet is observable in rearing trays, small amounts of larval diet should be added. Water should not be allowed to become murky as this can allow for scum formation. In rearing trays where scum formation is observed, it may be necessary to remove some of the water and film and add clean water to the rearing tray.

Approximately 5–6 d after mosquito eggs are hatched, pupae should begin to appear in the rearing tray. The males will pupate first followed by the females. These pupae will need to be transferred to an emergence chamber (for F0 populations) or into small cups if (for F1 or F2 populations). To pick pupae, cut the tip off a transfer pipette and so a pupa can fit inside the pipette. Using the pipette, pick the pupae from the larval rearing tray and transfer them into the appropriate container. Container mosquitoes will only remain in the pupal stage for 24–48 h and neglecting to pick pupae will result in adult mosquitoes emerging directly from the rearing tray.

Because F0 populations are field-collected eggs, you will not necessarily know the species that laid that egg. Therefore, it is necessary to sort the mosquitoes as they emerge. Fill the bottom of the emergence container partially with water and transfer pupae from the rearing trays into that emergence container. Replace the top to the emergence container and allow the mosquitoes to emerge. On a daily basis, emerged adult mosquitoes should be removed using an aspirator, identified, and transferred to an adult mosquito cage. It is possible to sight-identify most species that may be found in container habitats. However, if sight ID is not possible, mosquitoes can be knocked down by placing in a −20°C freezer for 60 s (Chen and Hillyer 2013) and identified quickly under a microscope before transferring them to the appropriate mosquito cage. If a key is necessary for identification, Darsie, Jr. and Ward (2005) can be used. Each adult cage should only contain one species of mosquito. The process of individually identifying mosquitoes from the F0 population will need to be done for every field-collected mosquito to ensure that the population used in assays is a single species. The CDC bottle bioassay is not intended to be used with mixed populations of mosquitoes.

When working with an F1 or F2 population, pupae can be transferred directly into a small cup with water in it. This cup can then be placed inside a rearing cage where the mosquitoes can emerge. Cotton soaked with 10% sucrose solution should also be provided in the cage and ensure there is enough surface area for several mosquitoes to feed at once and that it is not entirely soaked with the sucrose solution. Squeeze off the excess liquid, so it is moist but will not stick to the mosquito’s legs and bodies and result in mortality.

After emergence, mosquitoes will mate, and the females will be ready to bloodfeed 3–5 d after emergence. Bloodfeeding protocols will depend on your organization, but females should be allowed to feed until a majority have taken a complete bloodmeal. Allow 30–45 min for bloodfeeding before removing the blood source. Prior to bloodfeeding, mosquitoes can be sugar-starved (replace with water-soaked cotton) for several hours.

Some species, such as *Ae. aegypti* and *Ae. albopictus*, develop well in a laboratory setting and will readily bloodfeed through artificial systems or on live hosts, such as small mammals or chickens. However, other species, such as *Ae. japonicus* and *Ae. triseriatus*, can be more challenging to colonize. Their willingness to take bloodmeals in a laboratory setting is reduced compared to *Ae. aegypti* and *Ae. albopictus*. It is therefore more challenging to work with field populations of these species, but adjustments to time of bloodfeeding, bloodmeal type, feeding stimulants, and other factors can be manipulated to improve laboratory rearing success.

Approximately 2 d following the bloodmeal, oviposition paper should be placed inside the adult rearing cage. Fill a small cup with water and line the inside of the cup with germination paper. Once mosquitoes begin ovipositing, egg papers can be replaced every 2–3 d to prevent egg papers from becoming too crowded and not easily quantified. The same population of mosquitoes can be bloodfed multiple times to obtain more eggs. However, mortality will steadily increase as the mosquitoes age.

Populations beyond F2 should not be used for insecticide susceptibility assays as they may differ significantly phenotypically from the field population. Additionally, mosquitoes used in the CDC bottle bioassay should all be approximately the same age, ideally 3–5 d old and have never taken a bloodmeal.

### CDC Bottle Bioassay

Detailed protocols on preparing solutions for and conducting the CDC bottle bioassay can be found in CDC (2013, 2016, 2020). Before conducting the assay with a field population of interest, it is necessary to determine the diagnostic dose and time. The diagnostic time is defined as the time point at which a susceptible population of a species reaches 100% mortality. If a field population is fully susceptible to the insecticide being tested, 100% mortality at the diagnostic time is expected. While the CDC has provided guidelines for diagnostic times and doses, it is possible (and recommended especially with new species) to calibrate this in your laboratory.

The diagnostic dose is the concentration of insecticide that each bottle receives. It is recommended that stock solutions are created so that 1 ml of the stock can be added to the bottle to achieve the diagnostic dose. For example, if the diagnostic dose is 10 μg per bottle, stock solutions should be 10 μg/ml. Treating and coating a bottle with 1 ml of 10 μg/ml will yield the diagnostic dose of 10 μg per bottle.

Five 250-ml Wheaton bottles will need to be prepared for every AI or that is being evaluated. Four of these bottles will be treated with the AI and one bottle will serve as the control and will only be coated with acetone. Add 1 ml of the stock solution to the four treated bottles and 1 ml of acetone to the control bottle. Cap the bottle and evenly coat the interior by swirling the 1 ml in the bottom of the bottle. Then, turn the bottle on its side and tilt it back and forth, so the solution coats the bottle’s sides. Rotate the bottle as you tilt it back and forth and ensure that all surfaces of the bottle have been coated at least twice. Remove the cap from the bottles, lay them on their sides, and roll them on the table until all visible signs of acetone are gone.

While bottles can be used shortly following treatment and drying, allowing at least 1 h before conducting the bottle bioassay will allow any remaining acetone to evaporate. Bottles can be stored uncapped in a dark space during this time. Bottles can also be left overnight and used the following day. However, some AIs require the bottle bioassay to be conducted shortly following treatment. It is best to run bottles treated with naled and deltamethrin within 2 h of treating the bottles.

The bottles are now ready to be used in the CDC bottle bioassay. Twenty to twenty-five mixed sex mosquitoes will need to be aspirated into each bottle. It is recommended that both male and female mosquitoes be used in the assay because they equally contribute genes that may confer resistance to progeny (CDC 2020). Practicing the process of transferring mosquitoes from their cage to the test bottles prior to an official assay is suggested. When transferring mosquitoes to the bottles, do not let the aspirator touch the inside of the bottle.

Mosquitoes should be introduced to the control bottle first and then into the four treated bottles. It is important to introduce mosquitoes into the five bottles as quickly as possible, so dead or unhealthy/weak mosquitoes can be quickly identified. After mosquitoes are introduced to the bottle, a timer can be started and mortality is recorded at 0 (immediately after introduction), 5, 10, 15, 30, 45, 60, 75, 90, 105, and 120 min after introduction. Mosquitoes should be counted as dead if they are unable to right themselves during the mortality count. The movement of the bottle during counts may encourage live mosquitoes to fly and this will aid in taking the mortality count.

A sample data sheet for recording this information can be found in Appendix 3 of CDC (2013). As a recommendation, when mortality counts are being conducted, first roughly determine if there are more alive or dead mosquitoes and take a count of whichever there is less of. For example, the 45-min mortality count is being conducted and there are two mosquitoes flying around and the rest are dead. Record ‘2’ in the alive column on the data sheet. Once the assay has ended, and mosquitoes are counted, you will be able to determine the number that were dead at 45 min by subtracting 2 from the total number in the bottle.

After the 2-h assay, mosquitoes can be killed by freezing and then quantified for each bottle and recorded on the data sheet. However, if a 24-h mortality assessment is to be done, do not freeze mosquitoes after 2 h. Percent mortality can be calculated for each time point. If there was mortality in the control, Abbott’s formula should be used to correct the mortality (Abbott 1925). If mortality in the control is greater than 10%, the assay should be discarded.

The corrected mortality is calculated using the following formula: $\frac{\left(\%\;mortality\;in\;bottles\;with\;AI-\%\;mortality\;in\;control\;bottle\right)}{\left(100\;\%\;-\;\%\;mortality\;in\;control\;bottle\right)}\;\times \;\;\;100$. For example, if the mortality in the bottles with AI was 80% at the diagnostic time and was 3% in the control at the same time point, the corrected mortality would be $\frac{\left(80\;\%-3\%\right)}{\left(100\;\%-3\%\right)}\times 100\;\;=79.4\%$.

The insecticide susceptibility status is determined by the mortality of the population of interest at the diagnostic time. Greater than 97% mortality is classified as susceptible, 90–96% mortality as developing resistance, and less than 90% mortality as resistant. Follow-up testing based on these results can be conducted and are outlined in CDC (2016).

### 24-Hour Bottle Bioassay Addition

After the 2-h CDC bottle bioassay is complete, the assay can be extended to incorporate an optional 24-h addition. This enables recovery to be monitored once the mosquito has been removed from exposure. Recovery from knockdown has been noted previously and failing to incorporate a 24-h reading during an assay could allow resistance to be overlooked (Owusu et al. 2015, Richards et al. 2017).

Instead of placing mosquitoes in the freezer to be killed and quantified, mosquitoes from the bottle bioassay can be transferred to clean holding cages. If all mosquitoes are dead at the end of the 2-h assay, mosquitoes can simply be transferred by uncapping the bottle and dumping them into the holding cup. After transferring, the lid and tulle can be replaced. If there are still live mosquitoes at the end of the 2-h assay, a mechanical aspirator can be used to remove mosquitoes from the bottle and transferred into the clean holding cage. Mosquitoes can be transferred using the hole in the side of the paper cup and a piece of cotton should be used to close this hole once the transfer is complete.

During the holding period, mosquitoes should be provided with 10% sucrose solution. A cotton ball can be soaked with the sucrose solution and then squeezed out to remove excess liquid. If too much liquid is left in the cotton ball, it can drip into the holding cage and potentially result in additional mosquito mortality.

The 24-h mortality readings should be taken 24 h after the start of the CDC bottle bioassay. Mortality should be recorded in the same way it was recorded during the CDC bottle bioassay.

### Method Validation

To highlight the importance of the addition of the 24-h mortality reading to the CDC bottle bioassay and to validate the methodology described above, the 24-h addition was implemented using four different populations of *Ae. aegypti*. Two field populations of mosquitoes were collected from Pasco County, FL (Congress and Fox Hollow) using the collection protocols described above. The pyrethroid-susceptible laboratory strain, Orlando (ORL 1952; Pridgeon et al. 2008), and the pyrethroid-resistant laboratory strain, Puerto Rico (Estep et al. 2017) were also used for these assays. All mosquito populations were reared according to protocols described above.

CDC bottle bioassays were conducted using protocols described above and using diagnostic doses and times provided in Table 2. After the final reading was taken at 120 min for the CDC bottle bioassay, mosquitoes were transferred to clean holding containers and held until the 24-h mortality reading. Mortality was adjusted using Abbott’s formula.

**Table 2.** Diagnostic dose and time for AIs used in the CDC bottle bioassays

Chemical	Diagnostic dose (μg per bottle)	Diagnostic time (min)
Permethrin	43	15
Malathion	400	30
Deltamethrin	0.75	45
Naled	2.25	45
Etofenprox	12.5	30
Chlorpyrifos	20	45

Paired *t*-tests were conducted to compare the percent mortality of mosquitoes at the 2- and 24-h mortality reading. This was done for all mosquito populations in response to all AIs and for organophosphate and pyrethroid AIs. Analyses were performed using R statistical software (R Core Team 2019).

## Results

When AIs were grouped by chemical class, there was a significant change in the 2- and 24-h mortality for pyrethroid AIs (*t* = 6.5204, *P* < 0.001). Organophosphate AIs exhibited no change in mortality from the 2-h to the 24-h mortality reading.

**Fig. 6.**
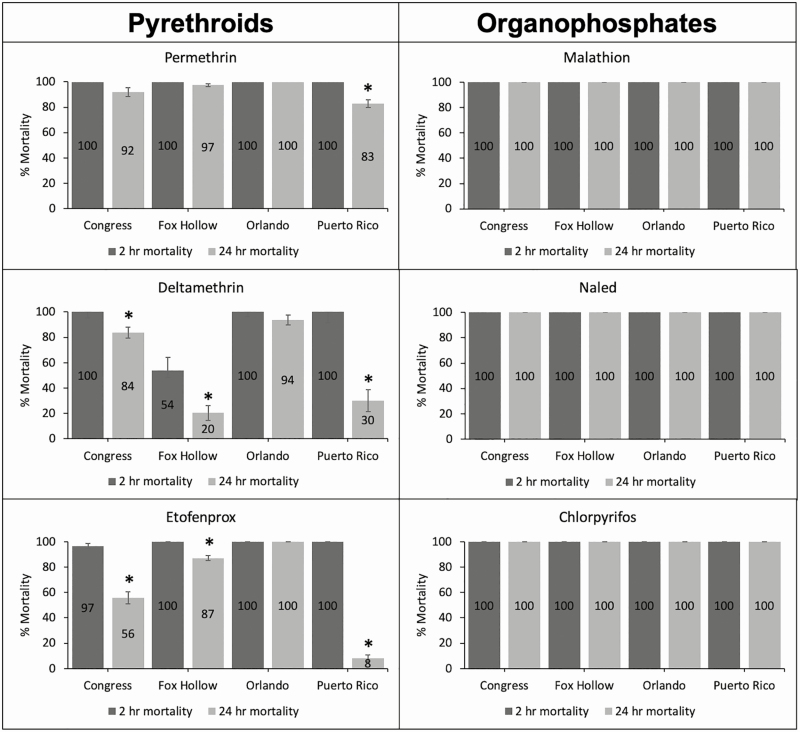
Response of two field populations (Congress and Fox Hollow), a pyrethroid-susceptible laboratory colony (Orlando), and pyrethroid-resistant laboratory colony (Puerto Rico) of *Aedes aegypti* to three pyrethroid and three organophosphate AIs. Dark gray bars indicate the percent mortality at the end of the CDC bottle bioassay (2-h mortality) and the light gray bars are the percent mortality after the mosquitoes were held in a clean cage for a 24-h reading. Asterisks indicate a significant difference in mortality between the 2- and 24-h mortality reading.

The Congress, Fox Hollow, Orlando, and Puerto Rico mosquito populations reached 100% mortality for all organophosphate AIs by the end of the 2-h CDC bottle bioassay and this was unchanged at the 24-h mortality reading (Fig. 6). In response to the pyrethroid AIs, all populations were perceived to have reached 100% mortality by the end of the 2-h assay with the exception of Fox Hollow in response to deltamethrin (54%) and Congress in response to etofenprox (97%). Additionally, there were significant differences in the mortality recorded at the 2- and 24-h mortality readings for Congress in response to deltamethrin (*t* = 3.7798, *P* = 0.03) and etofenprox (*t* = 8.3679, *P* = 0.004), Fox Hollow in response to deltamethrin (*t* = 7.6069, *P* = 0.005) and etofenprox (*t* = 6.3012, *P* = 0.008), and Puerto Rico in response to permethrin (*t* = 5.7756, *P* = 0.01), deltamethrin (*t* = 8.0668, *P* = 0.004), and etofenprox (*t* = 33.022, *P* < 0.001).

## Discussion

Mosquito rearing protocols are highly species-dependent and limited by the space that is available to rear them in. This protocol assumes access to a laboratory where temperature, humidity, and the light:dark cycle can be controlled. However, these resources are not always available to the groups that might benefit the most from having them. For example, budgets of local mosquito control programs vary widely. While it would be preferable for every group to conduct resistance monitoring in their region, many do not have the resources or expertise to accomplish this on a regular basis. However, the protocols described here can also be implemented by a centralized group, such as an academic institution, that may have the resources necessary to conduct laboratory rearing and resistance assays.

While a limited budget is somewhat of a disadvantage, the items listed in Table 1 are manageable for many programs or other organizations. Equipment that is already available can also be used, further reducing the cost. Additionally, items can be substituted with less expensive or more readily obtained materials. The materials needed to conduct the CDC bottle bioassay are relatively more expensive, but a bottle bioassay kit can be obtained by requesting an order form from USBottleAssayKit@cdc.gov.

Several protocols exist on how to rear *Aedes* mosquitoes in a laboratory setting (Gaffigan and Pecor 1997, Imam et al. 2014, Bei Resources 2016, Kauffman et al. 2017, Ote and Kanuka 2018). In many cases, learning how to collect and rear mosquitoes comes from experiential learning and tinkering with existing protocols. The slight differences between existing protocols and those presented here are due to sequential small changes that optimize the rearing conducted at the University of Florida, Florida Medical Entomology Laboratory. These protocols provide a new laboratory with a starting point and protocols to base rearing on, but they will inevitably be altered based on what works best for that particular laboratory with their particular populations of mosquitoes. Particularly, the protocols presented here are ideal for monitoring over a large geographic area, as container mosquitoes can be readily transported and are desiccation-resistant. The protocols presented here do not replace existing protocols, but rather add to an existing body of literature to aid in building basic mosquito-related skill sets.

The CDC bottle bioassay is a highly flexible assay because it allows for calibration, the addition of synergists, and the ability to identify some resistance mechanisms. It also allows for the evaluation of any AI (and synergist combinations) or formulated product, although the evaluation of formulated product using the CDC bottle bioassay is not recommended by the CDC. This may give it preference over other assays, such as the WHO assay. These two assays are not interchangeable but seem to agree on resistance status when a 24-h mortality is recorded (Owusu et al. 2015). Therefore, the 24-h addition to the CDC bottle bioassay enables monitoring for recovery and results in similar resistance classifications when compared to another widely used resistance detection method.

The addition of a 24-h mortality reading to the CDC bottle bioassay has been inconsistently done by various authors as an ad hoc addition to the assay (Aïzoun et al. 2013, Bagi et al. 2015, Denlinger et al. 2016, Mendoza 2016), but a formalized protocol has not previously been provided. The importance of having a 24-h mortality reading is highlighted in Fig. 6 where recovery is observed after exposure to some pyrethroids, but not after exposure to organophosphates. Recovery after removal from insecticide exposure was also observed in Bagi et al. (2015), Mendoza (2016), and Richards et al. (2017). Monitoring the recovery of mosquitoes beyond the 2-h cutoff of the CDC bottle bioassay reveals additional information about the resistance profile of mosquitoes, which allows for more informed control decisions to be made.

Collecting, rearing, and performing resistance monitoring with local populations of mosquitoes is a critical process in the control of invasive and/or vector species. Implementation of these protocols will improve the ability of public health personnel to control mosquitoes. Protocols provided here are a foundation for establishing a rearing program for container mosquitoes as well as resistance monitoring that incorporates a 24-h mortality reading.
